# Prevalence of Overweight and Obesity Among Adults and Their Association With Diabetes, Hypertension, and Dyslipidemia: A Comparative Cross-Sectional Study in Urban and Rural Areas of the Etawah District

**DOI:** 10.7759/cureus.86946

**Published:** 2025-06-29

**Authors:** Lokesh Raheja, Divyata Sachan, Prashant K Bajpai, Naresh P Singh, Ajai Kumar, Dhiraj K Srivastava, Sushil K Shukla

**Affiliations:** 1 Department of Community Medicine, Amar Shaheed Jodha Singh Attaiya Thakur Dariyao Singh Medical College, Fatehpur, IND; 2 Department of Community Medicine and Public Health, King George's Medical University, Lucknow, IND; 3 Department of Community Medicine, Uttar Pradesh University of Medical Sciences, Etawah, IND; 4 Department of Biochemistry, Uttar Pradesh University of Medical Sciences, Etawah, IND

**Keywords:** dyslipidemia, metabolic disorder, non-communicable diseases, obesity, overweight

## Abstract

Introduction

Obesity is not technically a terminal illness, yet it is a significant risk factor for numerous severe non-communicable diseases (NCDs). It is a condition characterized by excessive fat accumulation in the body, which can negatively impact health, leading to various complications and reduced life expectancy. In this study, we aim to estimate the prevalence of overweight and obesity and examine their association with diabetes, hypertension, and dyslipidemia. We also aim to determine the sociodemographic and clinical correlates of overweight and obesity, diabetes, hypertension, and dyslipidemia among adults in rural and urban areas of the Etawah district in Uttar Pradesh, India.

Methods

This cross-sectional study was conducted from January 2021 to June 2022. Study participants were selected using multistage random sampling. The study tool included a questionnaire based on the WHO STEPwise approach to NCD risk factor surveillance (STEPS). Descriptive analysis was performed using the Chi-square test and Student’s unpaired t-test. Multivariate regression analysis was conducted to assess the strength of the association.

Results

The prevalence of overweight/obesity was almost similar in rural and urban areas. In rural populations, body mass index (BMI) showed a statistically significant association with diabetes status, hypertension status, systolic blood pressure, diastolic blood pressure (DBP), serum cholesterol, triglycerides, and very-low-density lipoprotein cholesterol. However, in urban populations, BMI was significantly associated only with diabetes status and DBP. Overweight was found to be more prevalent in upper socio-economic status residents (1.67 (0.93 - 2.98)), smokers (2.02 (1.19 - 3.41)), hypertensives (3.03 (1.34 - 6.85)), and participants with dyslipidemia (1.79 (1.13 - 2.83)).

Conclusions

The present investigation revealed a significant prevalence of overweight and obesity in the Etawah district, a critical risk factor for non-communicable illnesses in both urban and rural settings, necessitating a robust public health strategy.

## Introduction

Obesity is a medical condition characterized by the excessive accumulation of body fat, which can adversely affect health, leading to various complications and reduced life expectancy [1]. The excess body fat may present as generalized obesity or central (abdominal) obesity. The anthropometric metrics often employed for evaluating obesity include body mass index (BMI) and waist circumference (WC). According to the Asia-Pacific guidelines for Southeast Asians by the World Health Organization (WHO), generalized obesity is defined as BMI ≥25 kg/m², overweight as BMI 23-24.9 kg/m², and abdominal obesity is defined as WC ≥90 cm (in males) and ≥80 cm (in females) [2].

Obesity and overweight have become significant public health concerns in both developed and developing countries [3,4]. Since 1975, the number of obese people worldwide has almost tripled. In 2016, approximately 1.9 billion individuals (39%) aged 18 and over were classified as overweight; moreover, 650 million (13%) of them were obese. Most people on Earth live in countries where overweight and obesity are more common causes of death than underweight [3]. In the 21st century, obesity has attained epidemic levels in India, with morbid obesity impacting 5% of the population. India is adhering to a pattern observed in other emerging nations that are progressively experiencing increased obesity rates [5]. According to the National Family Health Survey (NFHS-5) in India, the prevalence of overweight or obesity among women (BMI ≥ 25.0 kg/m²) is 24.0% (33.2% in urban and 14.7% in rural areas), and among men, it is 22.9% (29.8% in urban and 19.3% in rural areas), whereas its prevalence according to NFHS-4 was 20.6% and 18.9%, respectively, and its prevalence in Uttar Pradesh is 21.3% among women (30.6% in urban and 18.3% in rural areas) and 18.5% among men (24.9% in urban and 16.2% in rural areas) [6,7].

Obesity is not inherently a lethal condition; nonetheless, it serves as a risk factor for several significant non-communicable illnesses, namely dyslipidemia, hypertension, hyperglycemia, and a conglomeration of these conditions known as metabolic syndrome, which may even lead to death. Worldwide, about 58% of diabetes mellitus patients and 21% of ischemic heart disease patients have a BMI of more than 21 kg/m² [8-11]. Although avoidable, diabetes and hypertension rank among the top 10 leading causes of mortality worldwide. India is a developing nation experiencing a dual burden of undernutrition, attributed to poverty, and obesity, resulting from industrialization, fast urbanization, physical inactivity, and high-calorie foods associated with a sedentary lifestyle.

This study was undertaken to fill the gap in region-specific evidence from Etawah, where changing lifestyles, food habits, and social conditions differ from those in big cities. Understanding these local factors is crucial for designing public health strategies that effectively address the needs of this population. Thus, the present study was conducted to estimate the proportion of overweight/obesity, diabetes, hypertension, and dyslipidemia among adults, and to determine the association between overweight/obesity and diabetes, hypertension, and dyslipidemia among adults in urban and rural areas of the Etawah district.

## Materials and methods

This community-based cross-sectional study was conducted in eight villages in rural areas and eight wards in urban areas in Etawah district, Uttar Pradesh, India, from January 2021 to June 2022. The study participants were adults aged 18 years and above who provided informed written consent and had resided in the Etawah district for at least one year. Study participants with serious illness, pregnant or lactating females, and those suffering from any other acute or chronic illness apart from the diseases under investigation were excluded from the study. A multistage random sampling technique was used to enroll participants for the study.

The sample size was calculated using the following formula: $\text{Sample size} = \frac{\left[ Z_{1-\frac{\alpha}{2}} \right]^2 \left[ P_1 (1 - P_1) + P_2 (1 - P_2) \right]}{d^2}$, where Z_(1-α/2)_ is the standard normal deviate at a 95% confidence interval (CI).

Based on NFHS-5 (2019-2021, Uttar Pradesh), the estimated prevalence of overweight/obesity is 28% in urban areas and 17% in rural areas [7]. Hence, P₁ = 0.28, P₂ = 0.17, and the absolute error (d) is 0.11.

**Figure 1:**
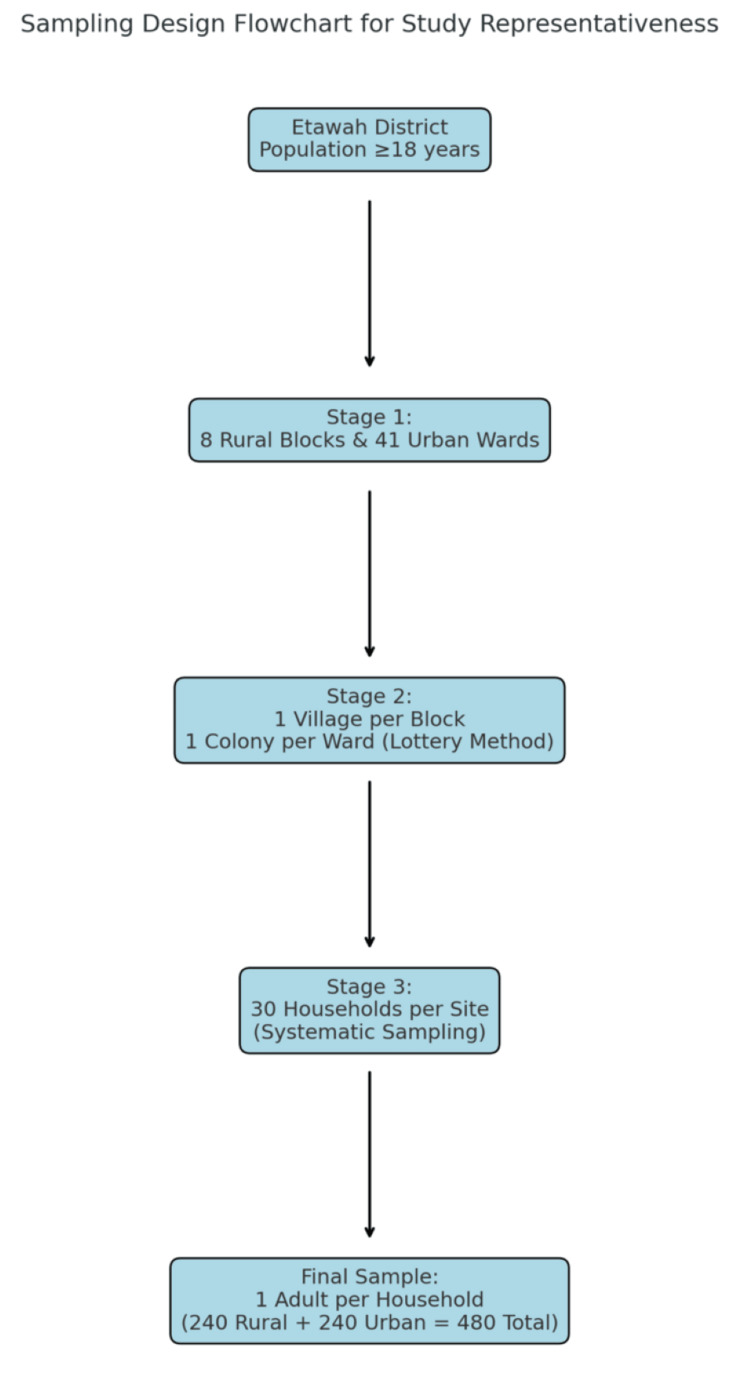
Sampling Design Flowchart for Study Representativeness

The minimum calculated sample size for each group was 109. After applying a design effect of 2.2, the adjusted sample size per group became 240 (109 × 2.2). Therefore, the total sample size comprised 480 participants (240 from rural and 240 from urban areas). Hence, 30 individuals from each block and each ward were enrolled to complete the sample size. The sampling design flowchart illustrating study representativeness is presented in Figure 1.

The study is based on the WHO STEPwise approach to NCD risk factor surveillance (STEPS) [12]. The steps in the study were as follows: Data Collection (STEP 1): The protocol developed by the WHO-STEPS program was adopted. A predesigned and pretested, semi-structured questionnaire (see Appendix 1) on identification data, socio-demographic variables (name, age, sex, occupation, and education), and non-communicable disease (NCD) risk factors (tobacco use, alcohol use, physical activity, and diet) was administered in the local language. Clinical Measurements (STEP 2): Height, weight, and blood pressure were measured using standardized instruments and procedures. Biochemical Measurements (STEP 3): Total cholesterol (TC), triglycerides (TG), high-density lipoprotein (HDL), low-density lipoprotein (LDL), very low-density lipoprotein (VLDL), and random blood sugar (RBS) were measured using laboratory tests.

Methodology

The targeted population was selected using multistage sampling from blocks and wards in Etawah, Uttar Pradesh. Data collection from rural areas proceeded as follows: Stage 1: A list of all the villages in the eight community development blocks in the rural areas of the Etawah district was procured. Stage 2: A lottery method randomly selected one village from each of the eight blocks. Stage 3: Because the adequate minimum sample size was 240, 30 households were selected from each randomly selected village of the eight blocks to ensure representation from all the community development blocks. This selection of households was done using a systematic random sampling technique, enrolling the nth sampling unit according to the sampling interval of the respective village. 

The point of initiation of enrolment started from landmarks in the villages, such as the Pradhan’s house, the Panchayat Bhavan, the overhead water tank, and the government primary school, and we moved in a left-sided direction from this initial landmark point to enroll the study units. If the selected house had more than one eligible participant after considering the inclusion and exclusion criteria, only one participant was enrolled using the lottery method. If there was no eligible participant in the randomly selected household, we moved to the next listed household within the same sampling interval. This process continued until we achieved the enrollment of the required sample of study subjects. A similar process was performed in all eight villages to achieve an effective sample size of 240.

Data collection from urban areas: Stage 1: From the list of 41 wards, eight wards were randomly selected by the lottery method. Stage 2: From the eight selected wards, one colony from each ward was also randomly selected using the lottery method. Stage 3: Then, participants were recruited from alternate houses in the selected colonies. If a house had more than one eligible study unit, the lottery method was used to select one among them. Enrollment of participants continued until 30 participants were enrolled after being found eligible as per the inclusion criteria. The same method was applied to participants in the other wards. A total of 240 participants were recruited from the urban areas of the Etawah district.

A questionnaire with sections for sociodemographic information, anthropometric measurements (height, weight, and blood pressure), and biochemical measurements (random blood glucose, total serum cholesterol, serum TG, HDL cholesterol, LDL cholesterol, and VLDL cholesterol) was completed during the first visit to the family. Height was measured using a pre-validated stadiometer to the nearest tenth of a centimeter, weight was measured using the ‘Dr Trust’ digital weighing machine (Dr Trust Healthcare Pvt. Ltd., Mumbai, India) in kilograms to the nearest hundred grams, and blood pressure was measured using the ‘Omron HEM-7120’ (Omron Corporation, Kyoto, Japan) automatic blood pressure monitor in millimeters of mercury (mmHg).

A blood sample was collected for RBS and lipid profile estimation. The study participants were pre-informed to maintain an overnight fasting of at least eight hours. Subsequently, their blood samples were collected in the morning and sent to the biochemistry laboratory of the tertiary health care facility. The blood collection procedure was performed by trained and qualified technicians, thus ensuring the fulfillment of recommended biosafety guidelines. Under aseptic conditions, intravenous blood was drawn from a peripheral vein (i.e., medial cubital vein). Specifically, 2 mL of blood was collected during the first visit for RBS in a grey vacutainer (containing sodium fluoride), and 2.5 mL of blood sample was collected on the scheduled visit in a red vacutainer (plain vial) for lipid profile assay. Immediately after collection, samples were temporarily stored in cold boxes until they were transferred to the central biochemistry laboratory. All biochemical assays were conducted by the same team of laboratory technicians, using identical methodology throughout the research period to prevent inter-operator differences. 

A fully automatic Biochemistry Analyser BA-400 (BioSystems, Barcelona, Spain) was used to analyze RBS by the glucose oxidase-peroxidase method, TC by the cholesterol esterase-oxidase-peroxidase-amidopyrine method, serum TG by the glycerol phosphate oxidase-peroxidase-amidopyrine method, and HDL cholesterol by the direct method with polyethylene glycol pre-treated enzymes. VLDL was calculated using the Friedewald formula, whereas LDL was calculated using the following formula: $\text{LDL} = \text{TC} - (\text{HDL} + \text{VLDL})$. 

The data thus collected (methodological and biochemical blood reports from the laboratory) were entered into a Microsoft Excel spreadsheet (Microsoft® Corp., Redmond, WA, USA) and scrutinized for completeness. Descriptive analysis of the data was done using proportions and means. The normality of the collected data was assessed using the Kolmogorov-Smirnov method, where p > 0.05 indicated a normal distribution. While assessing associations, if the dependent variable was categorical, a Chi-square test was performed irrespective of the distribution of data. However, for numerical dependent variables, an independent t-test or one-way ANOVA was performed for normally distributed data, and the Mann-Whitney U test or Kruskal-Wallis test was used for skewed data. Multivariate regression analysis was performed to calculate unadjusted and adjusted odds ratios. The analysis was conducted using IBM SPSS Statistics for Windows, Version 25 (Released 2017; IBM Corp., Armonk, NY, USA).

## Results

A statistically significant difference was observed in the distribution of participants between rural and urban areas based on age (p = 0.035), gender (p = 0.008), educational status (p < 0.001), occupational status (p = 0.004), socioeconomic status (p < 0.001), and smokeless tobacco use (p < 0.001). In rural areas, most participants were aged 40-49 years (22.9%), female (58.3%), illiterate (66%), and from the lower-middle class (44.2%). In contrast, urban participants were primarily aged 19-29 years (28.3%), male (53.8%), graduates (93%), and from the upper-middle class (28.7%). In both settings, homemakers formed the largest occupational group (47.9% in rural and 32.9% in urban areas). A majority of participants in both populations had an inactive lifestyle, with a statistically significant difference observed between the two groups (Table 1).

**Table 1:** Socio-Demographic and Behavioral Characteristics of Study Participants in Urban and Rural Areas (N = 480) * Statistically significant association at 95% CI (p < 0.05); **Modified BG Prasad classification (as of January 2022) [13]

Variable Name		Rural (N = 240), Number (%)	Urban (N = 240), Number (%)	p-value
Age (in Years)	19-29	48 (20.0%)	68 (28.3%)	0.035*
	30-39	42 (17.5%)	32 (13.3%)	
	40-49	55 (22.9%)	67 (27.9%)	
	50-59	40 (16.7%)	40 (16.7%)	
	60-69	40 (16.7%)	26 (10.8%)	
	≥70	15 (6.3%)	7 (2.9%)	
Sex	Male	100 (41.7%)	129 (53.8%)	0.008*
	Female	140 (58.3%)	111 (46.3%)	
Educational Status	Illiterate	66 (27.5%)	32 (13.3%)	<0.001*
	Just Literate (Primary)	53 (22.1%)	26 (10.8%)	
	High School	45 (18.8%)	44 (18.3%)	
	Intermediate School	28 (11.7%)	45 (18.8%)	
	Graduate and Above	48 (20.0%)	93 (38.8%)	
Occupational Status	Government Employee	7 (2.9%)	26 (10.8%)	0.004*
	Non-government Employee	19 (7.9%)	27 (11.3%)	
	Self Employed	64 (26.7%)	66 (27.5%)	
	Unemployed	13 (5.4%)	15 (6.3%)	
	Student	12 (5.0%)	16 (6.7%)	
	Homemaker	115 (47.9%)	79 (32.9%)	
	Retired	10 (4.2%)	11 (4.6%)	
Socioeconomic Status**	Upper	19 (7.9%)	47 (19.6%)	<0.001*
	Upper Middle	17 (7.1%)	69 (28.7%)	
	Middle	32 (13.3%)	59 (24.6%)	
	Lower Middle	106 (44.2%)	52 (21.7%)	
	Lower	66 (27.5%)	13 (5.4%)	
Tobacco User	Yes	46 (19.2%)	43 (17.9%)	0.725
	No	194 (80.8%)	197 (82.1%)	
Smokeless Tobacco Users	Yes	41 (17.2%)	11 (4.6%)	<0.001*
	No	198 (82.8%)	229 (95.4%)	
Use of Alcohol	Yes	42 (17.5%)	47 (19.6%)	0.557
	No	198 (82.5%)	193 (80.4%)	
Use of Fruits and Vegetables	Insufficient	225 (93.8%)	219 (91.3%)	0.298
	Sufficient	15 (6.3%)	21 (8.8%)	
Physical Activity	Vigorous Exercise	23 (9.6%)	11 (4.6%)	0.019*
	Moderate Exercise	74 (30.8%)	60 (25.0%)	
	Mild Exercise	49 (20.4%)	45 (18.8%)	
	Inactivity	94 (39.2%)	124 (51.7%)	

The prevalence of overweight/obesity, based on Quetelet’s Index (BMI), was nearly identical in rural areas at 37.9% (95% CI: 32.0-44.2) and in urban areas at 39.2% (95% CI: 33.2-45.5). However, according to WC, the prevalence of abdominal obesity was slightly lower than BMI-defined obesity in both rural areas, at 35.8% (95% CI: 30.0-42.0), and urban areas, at 37.5% (95% CI: 31.6-43.8). Additionally, the prevalence of diabetes was 7.9% (95% CI: 5.1-12.0) in urban and 4.2% (95% CI: 2.3-7.5) in rural areas; hypertension was 35.8% (95% CI: 30.0-42.0) in urban and 28.8% (95% CI: 23.4-34.8) in rural areas; and dyslipidemia (raised TC) was 27.1% (95% CI: 21.9-33.1) in urban and 26.7% (95% CI: 21.5-32.6) in rural areas. A statistically significant difference was observed in hypertension prevalence (p < 0.001) between rural and urban populations. However, no significant difference was found in BMI, WC, and diabetes prevalence between the two groups (Table 2).

**Table 2:** Comparison of Overweight/Obesity, Diabetes, Hypertension, and Dyslipidemia Status Among Study Participants According to Their Area of Residence * Statistically significant association at 95% CI (p < 0.05); ¨ADA classification; § Eighth Joint National Committee (JNC-8) RBS, Random Blood Sugar; HDL, High-Density Lipoprotein; LDL, Low-Density Lipoprotein; VLDL, Very Low-Density Lipoprotein

Variable Name		Rural (N = 240), Number (%)	Urban (N = 240), Number (%)	p-value
Quetelet’s Index (BMI in Kg/m²)	Normal/Underweight	149 (62.1%)	146 (60.8%)	0.778
	Overweight/Obese	91 (37.9%)	94 (39.2%)	
Waist Circumference	Normal (Males: <90 cm, Females: <80 cm)	154 (64.2%)	150 (62.5%)	0.705
	Raised (Males: ≥90 cm, Females: ≥80 cm)	86 (35.8%)	90 (37.5%)	
Diabetes Status (RBS in mg/dL)¨	Non-diabetics (<200)	230 (95.8%)	221 (92.1%)	0.085
	Diabetics (>200)	10 (4.2%)	19 (7.9%)	
Hypertension Status (mmHg)^§^	Normotensive (<120/80)	50 (20.8%)	11 (4.6%)	<0.001*
	Pre-hypertensives (≥120/80 - <140/90)	121 (50.4%)	143 (59.6%)	
	Hypertensives (≥140/90)	69 (28.8%)	86 (35.8%)	
Serum Triglycerides (mg/dL)	Normal (60-150)	130 (54.2%)	123 (51.2%)	0.522
	Raised (>150)	110 (45.8%)	117 (48.8%)	
Serum Cholesterol (mg/dL)	Normal (130-200)	176 (73.3%)	175 (72.9%)	0.918
	Raised (>200)	64 (26.7%)	65 (27.1%)	
HDL Cholesterol (mg/dL)	Normal (40-65)	225 (93.8%)	228 (95.0%)	0.522
	Low (<40)	15 (6.3%)	12 (5.0%)	
LDL Cholesterol (mg/dL)	Normal (up to 140)	218 (91.6%)	212 (89.1%)	0.352
	Raised (>140)	20 (8.4%)	26 (10.9%)	
VLDL Cholesterol (mg/dL)	Normal (12-30)	132 (55.9%)	124 (52.5%)	0.46
	Raised (>30)	104 (44.1%)	112 (47.5%)	

In both rural and urban areas, mean ± SD RBS levels were significantly higher in overweight/obese participants (Rural: 109.87 ± 56.40, Urban: 116.97 ± 52.01) compared to those with normal BMI (Rural: 88.87 ± 21.66, Urban: 98.74 ± 33.08). In rural areas, BMI was significantly associated with diabetes (p = 0.001), hypertension (p < 0.001), systolic blood pressure (p < 0.001), diastolic blood pressure (DBP) (p < 0.001), serum cholesterol (p = 0.009), TG (p < 0.001), and VLDL cholesterol (p < 0.001). However, in urban areas, only diabetes (p = 0.002) and DBP (p < 0.001) showed a significant association with BMI (Table 3).

**Table 3:** Association of Overweight/Obesity Status With Diabetes, Hypertension, and Biochemical Markers of the Lipid Profile Among Study Subjects in Rural and Urban Areas # Chi-square test applied; $ Student’s unpaired t-test; * Statistically significant association at 95% CI (p < 0.05) RBS, Random Blood Sugar; HDL, High-Density Lipoprotein; LDL, Low-Density Lipoprotein; VLDL, Very Low-Density Lipoprotein

Variable Name		Rural			Urban		
		BMI < 23 kg/m² (N = 149), Number (%)	BMI < 23 kg/m² (N = 91), Number (%)	p-value	BMI < 23 kg/m² (N = 149), Number (%)	BMI < 23 kg/m² (N = 91), Number (%)	p-value
Diabetes Status (RBS in mg/dL)	<200	146 (98.0%)	84 (92.3%)	0.001*#	139 (95.2%)	77 (81.9%)	0.002*
	≥200	3 (2.0%)	7 (7.7%)		7 (4.8%)	17 (18.1%)	
	Mean ± SD	88.87 ± 21.66	109.87 ± 56.40	<0.001*$	98.74 ± 33.08	116.97 ± 52.01	0.001*
Hypertension Status (mmHg)	Normotensive (<120/80)	45 (30.2%)	5 (5.5%)	<0.001*#	8 (5.5%)	3 (3.2%)	0.189#
	Pre-hypertensives (≥120/80 - <140/90)	72 (48.3%)	49 (53.8%)		92 (63.0%)	51 (54.3%)	
	Hypertensives (≥140/90)	32 (21.5%)	37 (40.7%)		46 (31.5%)	40 (42.6%)	
Systolic Blood Pressure (mmHg)	≥120	93 (62.4%)	85 (93.4%)	<0.001*#	133 (91.1%)	88 (93.6%)	0.480#
	<120	56 (37.6%)	6 (6.6%)		13 (8.9%)	6 (6.4%)	
	Mean ± SD	123.05 ± 16.97	131.05 ± 11.53	<0.001*$	129.44 ± 10.75	131.98 ± 11.26	0.081$
Diastolic Blood Pressure (mmHg)	≥80	79 (53.0%)	78 (85.7%)	<0.001*#	121 (82.9%)	88 (93.6%)	0.015*#
	<80	70 (47.0%)	13 (14.3%)		25 (17.1%)	6 (6.4%)	
	Mean ± SD	79.08 ± 9.84	85.81 ± 8.07	<0.001*$	85.42 ± 7.49	86.98 ± 6.77	0.105$
Serum Cholesterol (mg/dL)	>200	31 (20.8)	33 (36.3)	0.009*#	36 (24.7)	29 (30.9)	0.292#
	130-200	118 (79.2)	58 (63.7)		110 (75.3)	65 (69.1)	
	Mean ± SD	168.03 ± 41.93	197.18 ± 66.71	<0.001*$	176.67 ± 41.47	193.32 ± 66.49	0.180$
Serum Triglycerides (mg/dL)	>150	53 (35.6)	57 (62.6)	<0.001*#	70 (47.9)	47 (50.0)	0.756#
	60-150	96 (64.4)	34 (37.4)		76 (52.1)	47 (50.0)	
	Mean ± SD	156.29 ± 100.53	191.04 ± 83.00	0.006*$	168.68 ± 91.29	180.23 ± 87.11	0.331$
HDL Cholesterol (mg/dL)	>65	8 (5.4)	7 (7.7)	0.471#	7 (4.8)	5 (5.3)	0.856#
	40-65	141 (94.6)	84 (92.3)		139 (95.2)	89 (94.7)	
	Mean ± SD	48.26 ± 36.67	50.10 ± 10.68	0.642$	48.24 ± 10.53	51.11 ± 10.90	0.043*$
LDL Cholesterol (mg/dL)	>140	10 (6.8)	12 (13.0)	0.258#	15 (10.3)	11 (11.8)	0.720#
	Up to 140	137 (93.2)	81 (89.0)		130 (89.7)	82 (88.2)	
	Mean ± SD	91.75 ± 28.88	108.81 ± 62.52	0.005*$	95.20 ± 34.09	107.23 ± 60.48	0.051$
VLDL Cholesterol (mg/dL)	>30	47 (32.4)	57 (62.6)	<0.001*#	67 (46.5)	45 (48.9)	0.720#
	12-30	98 (67.6)	34 (37.4)		77 (53.5)	47 (51.1)	
	Mean ± SD	29.17 ± 15.51	38.26 ± 16.59	<0.001*$	34.78 ± 30.01	34.80 ± 15.285	0.994$

Among urban participants, smoking and physical activity status were the only sociodemographic or behavioral variables significantly associated with BMI. In rural areas, only physical activity status showed a significant association with BMI categories (p < 0.001) (Table 4).

**Table 4:** Association of Sociodemographic and Behavioral Factors of Study Subjects With Overweight/Obesity Status in Rural and Urban Areas # Chi-square test applied; * Statistically significant association at 95% CI (p < 0.005); ** Modified BG Prasad classification (as of Jan 2022) [13]

Variable Name		Rural			Urban		
		Normal (N = 149)	Overweight/Obesity (N = 91)	p-value^#^	Normal (N = 149)	Overweight/Obesity (N = 91)	p-value^#^
Age (in Years)	19-29	31 (20.8%)	17 (18.7%)	0.588	41 (28.1%)	27 (28.7%)	0.569
	30-39	25 (16.8%)	17 (18.7%)		15 (10.3%)	17 (18.1%)	
	40-49	29 (19.5%)	26 (28.6%)		42 (28.8%)	25 (26.6%)	
	50-59	27 (18.1%)	13 (14.3%)		25 (17.1%)	15 (16.0%)	
	60-69	26 (17.4%)	14 (15.4%)		18 (12.3%)	8 (8.5%)	
	≥70	11 (7.4%)	4 (4.4%)		5 (3.4%)	2 (2.1%)	
Sex	Male	58 (38.9%)	42 (46.2%)	0.270	76 (52.1%)	53 (56.4%)	0.512
	Female	91 (61.1%)	49 (53.8%)		70 (47.9%)	41 (43.6%)	
Educational Status	Illiterate	43 (28.9%)	23 (25.3%)	0.770	21 (14.4%)	11 (11.7%)	0.983
	Just Literate (Primary)	33 (22.1%)	20 (22.0%)		16 (11.0%)	10 (10.6%)	
	High School	30 (20.1%)	15 (16.5%)		26 (17.8%)	18 (19.1%)	
	Intermediate School	15 (10.1%)	13 (14.3%)		27 (18.5%)	18 (19.1%)	
	Graduate	28 (18.8%)	20 (22.0%)		56 (38.4%)	37 (39.4%)	
Occupational Status	Government Employee	1 (0.7%)	6 (6.6%)	0.209	14 (9.6%)	12 (12.8%)	0.220
	Non-government Employee	13 (8.7%)	6 (6.6%)		18 (12.3%)	9 (9.6%)	
	Self Employed	39 (26.2%)	25 (27.5%)		41 (28.1%)	25 (26.6%)	
	Unemployed	9 (6.0%)	4 (4.4%)		9 (6.2%)	6 (6.4%)	
	Student	8 (5.4%)	4 (4.4%)		8 (5.5%)	8 (8.5%)	
	Homemaker	74 (49.7%)	41 (45.1%)		53 (36.3%)	26 (27.7%)	
	Retired	5 (3.4%)	5 (5.5%)		3 (2.1%)	8 (8.5%)	
Socioeconomic Status**	Upper	8 (5.4%)	11 (12.1%)	0.338	25 (17.1%)	22 (23.4%)	0.208
	Upper Middle	10 (6.7%)	7 (7.7%)		39 (26.7%)	30 (31.9%)	
	Lower Middle	21 (14.1%)	11 (12.1%)		43 (29.5%)	16 (17.0%)	
	Upper Lower	65 (43.6%)	41 (45.1%)		30 (20.5%)	22 (23.4%)	
	Lower	45 (30.2%)	21 (23.9%)		9 (6.2%)	4 (4.3%)	
Current Smokers	Yes	27 (18.1%)	19 (20.9%)	0.598	19 (13.0%)	24 (25.5%)	0.014*
	No	122 (81.9%)	72 (79.1%)		127 (87.0%)	70 (74.5%)	
Current Smokeless Tobacco Users	Yes	29 (19.6%)	12 (13.2%)	0.202	8 (5.5%)	3 (3.2%)	0.408
	No	119 (80.4%)	79 (86.8%)		138 (94.5%)	91 (96.8%)	
Use of Alcohol	Yes	28 (18.8%)	14 (15.4%)	0.500	24 (16.4%)	23 (24.5%)	0.126
	No	121 (81.2%)	77 (84.6%)		122 (83.6%)	71 (75.5%)	
Use of Fruits and Vegetables	Insufficient	137 (91.9%)	88 (96.7%)	0.140	136 (93.2%)	83 (88.3%)	0.194
	Sufficient	12 (8.1%)	3 (3.3%)		10 (6.8%)	11 (11.7%)	
Physical Activity	Vigorous exercise	19 (12.8%)	4 (4.4%)	<0.001*	8 (5.5%)	3 (3.2%)	0.004*
	Moderate exercise	41 (27.5%)	33 (36.3%)		28 (19.2%)	30 (31.9%)	
	Mild exercise	41 (27.5%)	8 (8.8%)		25 (17.1%)	19 (20.2%)	
	Inactivity	48 (51.1%)	46 (48.9%)		85 (58.2%)	42 (44.7%)	

Participants from the upper socioeconomic class had 1.72 times higher odds of being overweight/obese (95% CI: 1.02-2.90, p = 0.04), smokers had 1.63 times higher odds (95% CI: 1.03-2.60, p = 0.037), hypertensive individuals had 4.84 times higher odds (95% CI: 2.24-10.4, p < 0.001), hypercholesterolemic individuals had 1.71 times higher odds (95% CI: 1.13-2.58, p = 0.010), and hypertriglyceridemic individuals had 1.79 times higher odds (95% CI: 1.24-2.60, p = 0.002) compared to their respective counterparts (Table 5).

**Table 5:** Univariate Regression Analysis to Determine the Strength of Association of BMI Categories With Demographic and Behavioral Factors, Diabetes, Hypertension, Hypercholesterolemia, and Hypertriglyceridemia * Statistically significant association at 95% CI (p < 0.005) “Reference” denotes the baseline or comparator category for each independent variable, against which the odds ratios (OR) of other categories are compared.

Independent Variables	Categories	Overweight/Obesity (BMI ≥ 23 kg/m²)		
		OR	95% CI	p-value
Age Group	19-59 years	1.43	0.87-2.34	0.153
	≥60 years (Reference)			
Gender	Male	1.27	0.88-1.83	0.206
	Female (Reference)			
Education	Educated	1.23	0.77-1.95	0.381
	Uneducated (Reference)			
Occupation	Housewife	0.75	0.51-1.09	0.138
	Others (Reference)			
Socio-Economic Status	Upper	1.72	1.02-2.90	0.041*
	Others (Reference)			
Servings of Fruits and Vegetables	Insufficient	0.98	0.49-1.97	0.965
	Sufficient (Reference)			
Smoking	Yes	1.63	1.03-2.60	0.037*
	No (Reference)			
Alcohol	Yes	1.16	0.73-1.86	0.515
	No (Reference)			
Smokeless Tobacco	Yes	0.61	0.32-1.51	0.128
	No (Reference)			
Physical Inactivity	Yes	1.10	0.76-1.59	0.595
	No (Reference)			
Diabetes	Diabetics	1.13	0.52-2.43	0.746
	Non-diabetics (Reference)			
Hypertension	Yes	4.84	2.24-10.4	<0.001*
	No (Reference)			
Cholesterol Status	Hypercholesterolemia	1.71	1.13-2.58	0.010*
	Normal (Reference)			
Triglyceride Status	Hypertriglyceridemia	1.79	1.24-2.60	0.002*
	Normal (Reference)			

After adjusting for significant variables in multivariate regression analysis, smoking, hypertension, hypercholesterolemia, and hypertriglyceridemia remained independent risk factors for overweight/obesity (BMI ≥ 23 kg/m²) (Table 6).

**Table 6:** Multivariate Regression Analysis to Determine Strength of Association of the BMI Categories With Demographic and Behavioral Factors, Hypertension, Hypercholesterolemia, and Hypertriglyceridemia * Statistically significant association at 95% CI (p < 0.005) “Reference” denotes the baseline or comparator category for each independent variable, against which the odds ratios (OR) of other categories are compared.

Independent Variables	Categories	Overweight/Obesity (BMI ≥ 23 kg/m²)		
		OR	95% CI	p-value
Socio-Economic Status	Upper	1.67	0.93-2.98	0.086
	Others (Reference)			
Smoking	Yes	2.02	1.19-3.41	0.009*
	No (Reference)			
Hypertension	Yes	3.03	1.34-6.85	0.008*
	No (Reference)			
Dyslipidemia	Hypercholesterolemia	1.79	1.13-2.83	0.013*
	Normal (Reference)			
Dyslipidemia	Hypertriglyceridemia	1.64	1.09-2.39	0.017*
	Normal (Reference)			

## Discussion

The present study found a marginal difference in the prevalence of overweight/obesity between urban (39.2%) and rural (37.9%) populations, suggesting a narrowing gap in the obesity burden across settings. This convergence may reflect changing lifestyles in rural areas, including increased consumption of calorie-dense foods and reduced physical activity. Similar patterns have been reported in previous studies by Tripathy et al., Logaraj et al., and Msyamboza et al., which also highlight the growing concern of rising obesity rates in rural populations - traditionally considered lower risk [14-16]. Globalization and urbanization have increasingly influenced rural lifestyles, leading to minimal differences between rural and urban regions, particularly in the Etawah area of Uttar Pradesh, where socioeconomic and lifestyle factors show little variation [17,18]. However, studies by Gupta et al. and Anjana et al. indicate considerable disparities in overweight and obesity rates, with urban populations exhibiting higher prevalence [17,18].

In the present study, the prevalence of diabetes was lower (4.2% rural and 7.9% urban) compared to studies conducted in Tamil Nadu, such as the ICMR-INDIAB multicentric study (7.8% rural and 13.7% urban), a study in Kancheepuram (21.9%), and another district-level study (11.2% rural and 23.6% urban) [19-21]. This discrepancy may be attributed to urbanization and shifting lifestyle patterns, such as increased sedentary behavior, higher intake of processed and energy-dense foods, and reduced physical activity, particularly in urban populations. These behavioral transitions, compounded by changes in occupational structures and greater access to unhealthy dietary options, may predispose individuals to metabolic disorders. Multivariate regression analysis further confirmed that overweight/obesity was independently associated with diabetes, underscoring the role of excess body weight as a key modifiable risk factor contributing to the rising burden of type 2 diabetes across both settings. In urban areas, this association was particularly notable, indicating that BMI remains an important factor even where obesity prevalence is relatively lower. With rising life expectancy and rapid urbanization, the burden of non-insulin-dependent diabetes mellitus (NIDDM) in India is expected to increase further.

The prevalence of hypertension in this study was higher in urban areas (35.8%) compared to rural areas (28.7%), indicating a notable urban-rural disparity. These findings exceed those reported in earlier studies conducted in Tamil Nadu (28% rural and 17% urban), Gujarat (29% urban and 15.4% rural), and Nagpur (15.4% rural), suggesting a growing burden of hypertension across both settings, particularly in semi-urbanizing regions like Etawah [9,10,15]. The higher urban prevalence may reflect factors such as increased psychological stress, dietary sodium intake, physical inactivity, and greater exposure to processed foods. A statistically significant difference (p < 0.001) in hypertension prevalence was observed between urban and rural populations in our study, aligning with observations made by Oommen et al. and Bhagyalaxmi et al. (both p < 0.001), who reported similar patterns of elevated urban risk. However, this trend was not reflected in the findings of the Integrated Disease Surveillance Programme (IDSP) survey, which reported a more uniform hypertension distribution across regions [10,20,22].

A significant association was observed between BMI and hypertension in the rural population (p < 0.001), whereas in the urban population, the association was not statistically significant (p = 0.189). The lack of a significant association between BMI and hypertension in urban areas may be due to a younger age distribution, better access to healthcare, or early antihypertensive treatment not captured in the study. Similar findings were reported in a study by Gothankar, conducted at UHTC, Pune, where a significant association (p < 0.05) between BMI and hypertension was noted [5]. Gelber et al., in a prospective cohort study of 4,920 individuals, demonstrated a strong correlation between increased BMI and hypertension [23]. Multivariate logistic regression analysis in this study confirmed that overweight/obesity (BMI ≥ 23 kg/m²) significantly increased the risk of hypertension (OR = 3.03, 95% CI: 1.34-6.85, p = 0.008). Similar findings were reported in a study by Saeed, where significant associations were observed (OR = 2.08, 95% CI: 1.49-2.90, p < 0.005) [24].

The proportion of hypercholesterolemia, hypertriglyceridemia, and high LDL was lower in this study compared to previous research. In the rural population, the prevalence was hypercholesterolemia: 26.7%, hypertriglyceridemia: 45.8%, and high LDL: 8.4%, whereas in urban areas, the prevalence was hypercholesterolemia: 27.1%, hypertriglyceridemia: 48.8%, and high LDL: 10.9%. These rates were lower than those reported by Oommen et al., who observed higher hypercholesterolemia and hypertriglyceridemia prevalence in both rural (34.9% and 16.0%) and urban (44.3% and 20.7%) areas of Tamil Nadu. Similarly, in Kerala, the prevalence was higher (rural: hypercholesterolemia 56.0%, hypertriglyceridemia 18.8%; urban: hypercholesterolemia 61.0%, hypertriglyceridemia 21.9%), and in Delhi, hypercholesterolemia was 37.5%, and hypertriglyceridemia was 40.5% [20,25,26].

Statistically significant associations were observed between serum TG and overweight/obesity, but only in rural subjects. Multiple regression analysis further confirmed that hypercholesterolemia increased the odds of overweight/obesity (BMI ≥ 23 kg/m²) by 1.79 times (95% CI: 1.13-2.83, p = 0.013), which differed from studies conducted by Oommen et al. and Thankappan et al. [20,25]. Additionally, the presence of hypertriglyceridemia significantly increased the odds of overweight/obesity (OR = 1.64, 95% CI: 1.09-2.39, p = 0.017), consistent with previous studies by Oommen et al. (OR = 1.27, 95% CI: 1.06-1.51, p < 0.05) and Thankappan et al. (OR = 2.45, 95% CI: 1.72-3.50, p < 0.05) [20,25].

Limitations

The STEPS methodology provides standardized data on key modifiable risk factors through population-based surveys that do not require sophisticated technology. However, it does not assess body fat percentage, dietary fat, salt intake, total energy consumption, or physical activity using objective methods such as pedometers or accelerometers. A more comprehensive understanding of these associations would be possible with additional data on these parameters. The sampling units per block should have been increased to improve representativeness and the generalizability of the results. Since the current medical status of participants regarding hypertension, diabetes, and obesity was not clinically verified, those with a known medical history may have responded with greater awareness and accuracy compared to participants without any diagnosed condition, thus introducing information bias. This could have been prevented by excluding participants with a medical history of the diseases in question. The study did not capture detailed lifestyle factors such as dietary habits, occupational activity, and healthcare access, which may partly explain the observed urban-rural differences in BMI-NCD associations. Although this study analyzed diabetes, hypertension, and dyslipidemia as separate entities, future research should consider exploring metabolic syndrome as an integrated outcome to better understand the interrelated burden of cardiometabolic risk.

Strengths

The strengths of this study include the use of a geographically and demographically representative sample (n = 480), selected through multistage random sampling from all rural blocks and urban wards of Etawah district, and an analysis that accounted for the complex survey design. The wide range of variables examined provides a comprehensive assessment of these factors in the Uttar Pradesh population. Additionally, the high response rate reduces the risk of selection bias. The study adhered to WHO-standardized protocols, ensuring high data quality through rigorous training of data collection personnel, pre-study testing of procedures, and continuous monitoring.

## Conclusions

The present investigation revealed a significant prevalence of overweight and obesity in the Etawah district, a critical risk factor for non-communicable illnesses in both urban and rural settings, necessitating a robust public health strategy. It is essential to implement a surveillance system at the community level to monitor, assess, and guide policies and programs. To mitigate modifiable risk factors, measures such as tobacco regulation, the production and distribution of nutritious meals, the regulation of unhealthy foods, and urban design to encourage physical activity must be executed. The WHO STEPS method provides a framework for initiating NCD surveillance, facilitating the creation of a flexible, progressively extensive, and intricate surveillance system tailored to local requirements and resources. The study’s findings underscore the necessity for diverse treatments and strategies to mitigate risk factors associated with non-communicable illnesses in both rural and urban settings.

There is an urgent need to enhance awareness of risk factors for chronic NCDs within the general population. This can primarily be achieved through comprehensive information, education, and communication campaigns employing multifaceted strategies aimed at educating the younger generation - including children, adolescents, and adults - to prevent the early onset of smoking and alcohol consumption. The emphasis should primarily be on lifestyle change, early diagnosis and treatment through the identification of risk factors, and the prevention and management of problems by consistent medication and follow-up with qualified health care professionals. The media plays a crucial role in disseminating awareness through many platforms, including television, radio, and print media. The involvement of lawmakers, health programs, and laws is also crucial. Emphasis should be placed on the adequate coverage and execution of the National Programme for Prevention and Control of Non-communicable Diseases. The initiation of NCD surveillance is aimed at ensuring data comparability across time and across various sites.

## Appendices

Appendix 1: Working proforma

Prevalence of Overweight and Obesity Among Adults and Their Association With Diabetes, Hypertension, and Dyslipidemia: A Comparative Cross-Sectional Study in Urban and Rural Areas of the Etawah District

Step 1: Socio-demographic profile and behavioral factors

**Table 7:** Socio Demographic Profile and Behavioral Factors

S. No.	Questions		Response
A) Socio-demographic profile			
1	Interviewer ID		
2	Date of Interview		
3	First Name		
4	Family Surname		
5	Contact/Phone no.		
6	Gender		
	a.	Male	
	b.	Female	
7	Date of Birth		
8	Age (in years)		
9	How many years have you spent in school or full time study?		
10	Highest level of education you have completed?		
	a.	Illiterate	
	b.	Just Literate (Primary)	
	c.	High School	
	d.	Intermediate School	
	e.	Graduate	
11	Religion		
	a.	Hindu	
	b.	Muslim	
	c.	Sikh	
	d.	Christian	
	e.	Others	
12	Marital Status		
	a.	Married	
	b.	Widowed	
	c.	Separated	
	d.	Un-married	
13	Occupation		
	a.	Govt employee	
	b.	Non Govt employee	
	c.	Self employee	
	d.	Non paid	
	e.	Student	
	f.	Homemaker	
	g.	Retired	
	h.	Unemployed	
14	Number of family members		
15	How many people older than you live in your household		
16	Total monthly income of family from all sources (Rs.)		
17	Socioeconomic status of family according to Modified BG Prasad Scale		
	a.	Upper class- Rs6346 and above	
	b.	Upper middle - Rs3173-6345	
	c.	Lower middle- Rs1904-3172	
	d.	Upper lower- Rs952-1903	
	e.	Lower- Rs<951	
B) Behavioral factors			
Smoking habit			
1	Do you currently smoke any tobacco products, such as cigarettes, cigars or pipes?		
	a.	Yes	
	b.	No	
2	How old you were when you first started smoking? (For current smokers)		
3	On an average, how many of the following products do you smoke each day/week? (IF LESS THAN DAILY, RECORD WEEKLY)		
						Daily/Weekly
				a.	Manufactured cigarettes	
	b.	Hand rolled cigarettes	
	c.	Pipes full of tobacco	
	d.	Cigars/cheroots/cigarillos	
	e.	Number of shisha sessions	
	f.	Bidis	
	g.	Chillam	
	h.	Others	
	4	During the past 12 months, have you tried to stop smoking?		
		a.	Yes	
b.		No	
5	Do you currently use smokeless tobacco products daily?		
	a.	Yes	
	b.	No	
6	On an average, how many times a day/week do you use? (IF LESS THAN DAILY, RECORD WEEKLY) For current users of smokeless tobacco only.		
						Daily/Weekly
	a.	Snuff, by mouth	
	b.	Snuff, by nose	
	c.	Chewing tobacco	
	d.	Betel, quid	
	e.	Others	
	Alcohol consumption			
7	Have you ever consumed alcohol such as beer, wine, spirits, or any other local brand?		
	a.	Yes	
	b.	No	
8	During the past 12 months, how frequently have you consumed at least one standard alcoholic drink?		
	a.	Daily	
	b.	5-6 days per week	
	c.	3-4 days per week	
	d.	1-2 days per week	
	e.	1-3 days per month	
	f.	Less than once a month	
Dietary habits			
9	In a typical week, on how many days do you eat fruit?		
10	How many servings of fruit do you eat on one of those days?		
11	In a typical week, on how many days do you eat vegetables?		
12	How many servings of vegetables do you eat on one of those days?		
13	How often do you add salt or a salty sauce such as soya sauce to your food right before you eat it or as you are eating it?		
	a.	Always	
	b.	Often	
	c.	Sometimes	
	d.	Rarely	
	e.	Never	
14	How much salt or salty sauce do you think you consume?		
	a.	Far too much	
	b.	Too much	
	c.	Just the right moment	
	d.	Too little	
	e.	Far too little	
	f.	Don’t know	
15	How important to you is lowering the salt intake in your diet?		
	a.	Very important	
	b.	Somewhat important	
	c.	Not at all important	
	d.	Don’t know	
16	Do you think that too much salt or salty sauce in your diet could cause a health problem?		
	a.	Yes	
	b.	No	
17	What type of oil or fat is often used for meal preparation in your household?		
	a.	Vegetable oil	
	b.	Lard or suet	
	c.	Butter or ghee	
	d.	Margarine	
	e.	Other	
	f.	None in particular	
	g.	None used	
	h.	Dont know	
Physical activity			
18	Type of physical activity you are involved in:		
	a.	Vigorous exercise (regular) or strenuous (manual) work at home/work	
	b.	Moderate exercise (regular) or moderate physical activity at home/work	
	c.	Mild exercise (regular) or mild physical activity at home/work	
	d.	No exercise and sedentary activities at home/work	
History of raised blood pressure			
19	Have you ever had your blood pressure measured by a doctor or other health worker?		
	a.	Yes	
	b.	No	
20	In the past two weeks, have you taken any drugs (medications) for raised blood pressure prescribed by a doctor or other health worker?		
	a.	Yes	
	b.	No	
History of diabetes			
21	Have you ever had your blood sugar measured by a doctor or other health worker?		
	a.	Yes	
	b.	No	
22	In the past two weeks, have you taken any drugs (medication) for diabetes prescribed by a doctor or other health worker?		
	a.	Yes	
	b.	No	
History of raised cholesterol			
23	Have you ever had your cholesterol (ſat levels in your blood) measured by a doctor or other health worker?		
	a.	Yes	
	b.	No	
24	In the past two weeks, have you taken any oral treatment (medication) for raised total cholesterol prescribed by a doctor or other health worker?		
	a.	Yes	
	b.	No	

Step 2: Anthropometric measurements

**Table 8:** Anthropometric Measurements

S. No.	Parameters	Measurements
1.	Weight (kg)	
2.	Height (cm)	
3.	Total Skin fold thickness (mm) at 4 sites	
4.	Waist circumference (cm)	
5.	Hip-circumference (cm)	
6.	Waist-hip ratio	
7.	Waist-height ratio	
8.	Body mass index (kg/m^2^)	
9.	Systolic blood pressure (mm/Hg)	
10.	Diastolic blood pressure (mm/Hg)	
11.	BROCA's Index	
12.	PONDRAL Index	

Step 3: Biochemical investigations

**Table 9:** Biochemical Investigations

S. No.	Parameters	Response
1.	Technician ID	
2.	Device ID	
3.	Time And Date of blood specimen taken (24 hours clock)	
4.	Random blood glucose (mg/dl)	
5.	Today, have you taken insulin or other drugs (medications) that have been prescribed by a doctor or other health worker for raised blood glucose?	
6.	Blood lipid device ID.	
7.	Total cholesterol	
8.	During the past two weeks, have you been treated for raised cholesterol with drugs (medications) prescribed by a doctor or other health worker?	
9.	Triglycerides Levels	
10.	HDL Cholesterol Levels	
11.	LDL Cholesterol Levels	

## References

[1] Haslam DW, James WP (2005). Obesity. Lancet.

[2] (2025). The Asia Pacific perspective: redefining obesity and its treatment. https://iris.who.int/bitstream/handle/10665/206936/0957708211_eng.pdf.

[3] NewsRoom NewsRoom (2025). Obesity. World Health Organization (Home/Newsroom/Facts in pictures/Detail/Obesity).

[4] Ahirwar R, Mondal PR (2019). Prevalence of obesity in India: a systematic review. Diabetes Metab Syndr.

[5] Gothankar JS (2011). Prevalence of obesity and its associated comorbidities amongst adults. Natl J Community Med.

[6] (2025). National Family Health Survey 5 fact sheet India. https://mohfw.gov.in/sites/default/files/NFHS-5_Phase-II_0.pdf.

[7] Ministry of Health and Family Welfare GoI (2025). National Family Health Survey (NFHS-5), Uttar Pradesh Page 151. National Family Health Survey (NFHS-5), Uttar Pradesh.

[8] Inamdar A (2022). Association of body mass index with systolic and diastolic blood pressure in rural Indians. Eur Heart J.

[9] Bhardwaj SD, Shewte MK, Bhatkule PR, Khadse JR (2012). Prevalence of risk factors for non-communicable disease in a rural area of Nagpur district, Maharashtra - a WHO STEP wise approach. Int J Biol Med Res.

[10] Bhagyalaxmi A, Atul T, Shikha J (2013). Prevalence of risk factors of non-communicable diseases in a district of Gujarat, India. J Health Popul Nutr.

[11] Okpechi IG, Chukwuonye II, Tiffin N, Madukwe OO, Onyeonoro UU, Umeizudike TI, Ogah OS (2013). Blood pressure gradients and cardiovascular risk factors in urban and rural populations in Abia State South Eastern Nigeria using the WHO STEPwise approach. PLoS One.

[12] (2025). STEPwise approach to NCD risk factor surveillance (STEPS). https://www.who.int/teams/noncommunicable-diseases/surveillance/systems-tools/steps.

[13] Pentapati SSK, Debnath DJ (2023). Updated BG Prasad's classification for the year 2022. J Family Med Prim Care.

[14] Tripathy JP, Thakur JS, Jeet G, Jain S (2017). Prevalence and determinants of comorbid diabetes and hypertension: evidence from non-communicable disease risk factor STEPS survey, India. Diabetes Metab Syndr.

[15] Logaraj M, Balaji R, John KR, Kumar S, Hegde B (2014). Comparative study on risk factors for cardiovascular diseases between urban and rural population in Tamil Nadu. Natl J Res Community Med.

[16] Msyamboza KP, Ngwira B, Dzowela T, Mvula C, Kathyola D, Harries AD, Bowie C (2011). The burden of selected chronic non-communicable diseases and their risk factors in Malawi: nationwide STEPS survey. PLoS One.

[17] Gupta V, Yadav K, Anand K (2010). Patterns of tobacco use across rural, urban, and urban-slum populations in a north Indian community. Indian J Community Med.

[18] Anjana RM, Pradeepa R, Deepa M (2011). Prevalence of diabetes and prediabetes (impaired fasting glucose and/or impaired glucose tolerance) in urban and rural India: phase I results of the Indian Council of Medical Research-INdia DIABetes (ICMR-INDIAB) study. Diabetologia.

[19] Vijayakarthikeyan M, Krishnakumar J, Umadevi R (2017). Cross-sectional study on the prevalence of risk factors for non-communicable disease in a rural area of Kancheepuram, Tamil Nadu. Int J Community Med Public Health.

[20] Oommen AM, Abraham VJ, George K, Jose VJ (2016). Prevalence of risk factors for non-communicable diseases in rural & urban Tamil Nadu. Indian J Med Res.

[21] Samuel P, Antonisamy B, Raghupathy P, Richard J, Fall CH (2012). Socio-economic status and cardiovascular risk factors in rural and urban areas of Vellore, Tamil Nadu, South India. Int J Epidemiol.

[22] National Institute of Medical Statistics (2009). Risk Factors Survey, Indian Phase-I States (2007-2008). National Institute of Medical Statistics. IDSP Non-Communicable Disease Risk Factors Survey, Phase-I States of India, 2007-08.

[23] Gelber RP, Gaziano JM, Manson JE, Buring JE, Sesso HD (2007). A prospective study of body mass index and the risk of developing hypertension in men. Am J Hypertens.

[24] Saeed KM (2013). Prevalence of risk factors for non-communicable diseases in the adult population of urban areas in Kabul city, Afghanistan. Cent Asian J Glob Health.

[25] Thankappan K, Shah B, Mathur P (2010). Risk factor profile for chronic non-communicable diseases: results of a community-based study in Kerala, India. Indian J Med Res.

[26] Garg A, Anand T, Sharma U, Kishore J, Chakraborty M, Ray PC, Ingle GK (2014). Prevalence of risk factors for chronic non-communicable diseases using WHO steps approach in an adult population in Delhi. J Family Med Prim Care.

